# Phase-separation: a possible new layer for transcriptional regulation by glucocorticoid receptor

**DOI:** 10.3389/fendo.2023.1160238

**Published:** 2023-04-14

**Authors:** Ester da Silva Pinheiro, André Maciel Preato, Tamirez Villas Boas Petrucci, Lucas Souza dos Santos, Isaias Glezer

**Affiliations:** Department of Biochemistry, Escola Paulista de Medicina, Universidade Federal de São Paulo, São Paulo, Brazil

**Keywords:** immune response, liquid-liquid phase separation (LLPS), steroid receptors, transcription control, transregulation

## Abstract

Glucocorticoids (GCs) are hormones involved in circadian adaptation and stress response, and it is also noteworthy that these steroidal molecules present potent anti-inflammatory action through GC receptors (GR). Upon ligand-mediated activation, GR translocates to the nucleus, and regulates gene expression related to metabolism, acute-phase response and innate immune response. GR field of research has evolved considerably in the last decades, providing varied mechanisms that contributed to the understanding of transcriptional regulation and also impacted drug design for treating inflammatory diseases. Liquid-liquid phase separation (LLPS) in cellular processes represents a recent topic in biology that conceptualizes membraneless organelles and microenvironments that promote, or inhibit, chemical reactions and interactions of protein or nucleic acids. The formation of these molecular condensates has been implicated in gene expression control, and recent evidence shows that GR and other steroid receptors can nucleate phase separation (PS). Here we briefly review the varied mechanisms of transcriptional control by GR, which are largely studied in the context of inflammation, and further present how PS can be involved in the control of gene expression. Lastly, we consider how the reported advances on LLPS during transcription control, specially for steroid hormone receptors, could impact the different modalities of GR action on gene expression, adding a new plausible molecular event in glucocorticoid signal transduction.

## Introduction

Glucocorticoids (GCs) are steroid hormones that are physiologically synthesized and released according to stress stimuli and the circadian cycle. Cortisol in humans, and corticosterone in rodents, are considered the main hormones produced by the adrenal cortex that regulate metabolism and immune responses. Potent anti-inflammatory responses are mediated through the GC receptor (GR) (1–3). While GRs mediate crucial homeostatic control in higher vertebrates, the fact that therapeutic GR agonists are employed as potent anti-inflammatory drugs cannot be neglected, for instance Dexamethasone played a significant role in the treatment of COVID-19 critical patients worldwide. GR activation by steroidal anti-inflammatory drugs limit key cytokines production, including IL-1β, TNF-α and IL-6 (4–12). GCs are cell membrane permeable and bind to GR in the cytosol, leading to nuclear translocation of the ligand-receptor complex, where GR binds to DNA and nuclear proteins, regulating gene expression (7, 13, 14). It was recently described that GR, when interacting with chromatin, forms condensates, which might be related to liquid-liquid phase separation (LLPS) (15, 16).

The concept of LLPS has already been studied in the field of biology for some years (17–21), and it is the main type of phase-separation (PS) characterized in cell biology. This thermodynamic process consists of separating into two coexisting liquid phases, one dense (condensates) and the other dilute. It is well described that LLPS potentiates intracellular reactions, hijacks molecules or promotes molecular complexes. Recently, this process has been linked to transcriptional control and consequently impacts gene expression (22). In the steroid receptor (SR) pathways, this proposal has already been analyzed. *In vivo* and *in vitro* studies have shown that LLPS is not formed upon deletion of certain domains of these NRs (15, 23).

Transcriptional regulation caused by LLPS in GR pathways has not yet been analyzed in depth. Here we review the key elements of GR control on gene expression, which is predominantly studied in an inflammatory context, and select information associated with the participation of PS into molecular events that determine transcription control by SRs. Finally, we propose analogous modes of LLPS events that could contribute to the regulation of proinflammatory responses through GR. The aim of this short review is to outline the major molecular events that take place during varied modes of GR action on gene expression, and to suggest a place for LLPS in some of these key processes.

## Glucocorticoid receptors

**Figure 1:**
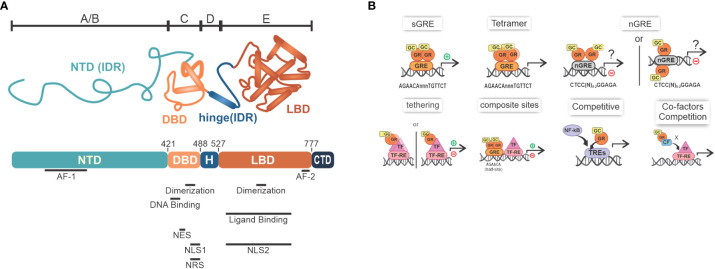
GR overview. **(A)** The GR structure containing NTD, or amino-terminal A/B region, that has varied sequences known as IDR, along with the largest transactivation site called AF-1, responsible for the interaction with coactivators, chromatin modulators and the transcription machinery; C region, which comprises the region that enables dimerization mediated by the presence of two zinc fingers type 2, in addition to the DBD, which recognizes GREs in DNA; D region (i.e., variable hinge region) that contains the nuclear localization signal sequences; and E region with the LBD, where there is a second ligand-dependent AF-2 activation site and regions important for dimerization, transactivation and stability maintenance during its inactive stage, which is accomplished by the interaction with HSPs; C-terminal regions differ between *α* and *β* isoforms [adapted from (23)]; **(B)** Main mechanisms of transcriptional modulation associated with GR: sGRE - main mechanism mediated by the interaction of GR dimers with GRE sites, which promotes transcription of target genes; nGRE - a transcription inhibitory (negative) site that GR binds as a monomer; Tetramer- GR might also exist as a tetramer; Tethering - protein-protein interaction forming a GR+TF dimer that can induce or inhibit gene expression (please note that GR binding to DNA has been described for this mechanism; see main text for detail); Composite sites - GRE sites located close to TF-RE sites that can promote or repress gene transcription; GR can compete with other TFs for binding to DNA, for example NF-κB and AP-1; Co-factors competition- GR can also compete with co-factors required for transcription. GR, glucocorticoid receptor; NTD, N-terminal domain; IDRs, intrinsically disordered regions; AF-1/2, activation function 1 or 2; DBD, DNA-bind domain; GRE, glucocorticoid response elements; LBD, Ligand-binding domain; HSPs, heat shock proteins; sGRE, GRE sequence; nGRE, negative GRE; TF-RE, transcription factors response elements; GCs, glucocorticoid.

Human GR (hGR; official symbol: NR3C1), such as main isoforms *α* and *β*, are part of the NR superfamily comprised by ligand-induced transcription factors that can transrepress (–) or transactivate different genes. Prototypical hGR*α* is composed of multiple domains, which, altogether, determine the concerted varied molecular interactions induced by GC signaling (**Figure 1A**) (24, 25). Once GCs become available in the cytoplasm and interact with GR coupled with HSP70, HSP90, p23 and FKBP51, profound changes in the protein complex promote the exposure of the two nuclear localization sequences present in GR. This enables GR translocation to the nucleus, which was thought to migrate in its dissociated form, but recent evidence suggests that the chaperone complex is necessary for efficient translocation [reviewed in (13)]. In this compartment, canonical GR activity on gene expression through DNA binding is mediated by the formation of a GR homodimer (GR+GR), although tetrameric binding has also been suggested (26) (**Figure 1B**). After binding to GRE sites, GR act as a transcription factor (TF) and recruits primary cofactors. In consequence, p160 superfamily member steroid receptor coactivator-1 [SRC-1; a.k.a. nuclear receptor coactivator-1 (NCOA1)] coordinates the clustering of secondary coactivators NCOA2 and p300. Their counterparts, cAMP-responsive element-binding protein (CREB)-binding protein (CBP), and CBP/p300 associated factor (pCAF) are recruited, playing significant roles in chromatin remodeling, transcriptional initiation complex recruitment and RNA polymerase II activation. Examples of canonical simple GRE transcribed genes through GR homodimer binding that downregulate inflammation include: *ANXA1* (Lipocortin I), *NFKBIA* (IκBα), *DUSP1* (MKP-1), *TSC22D3* (GILZ), and *ZFP36* (TPP). Coactivators recruited by GR dimers (e.g., p300/CBP) are also coactivators of TFs of pro-inflammatory pathways such as nuclear factor (NF)-*κ*B and activator protein (AP)-1. Thus, a second modulation mechanism attributed to GR would be to compete for the recruitment of coactivators, leading to lower expression of genes associated with these other TFs (27–32). The contribution of these mechanisms to GCs anti-inflammatory actions is significant, but often outshined by direct interference with NF-*κ*B and AP-1 transcription factors.

From the perspective of mechanisms of repression/inhibition of gene expression, initial evidence indicated that GR binds to negative GREs (nGREs) sequences, which are inverted repeat sequences where GR monomers are arranged in a head-to-tail orientation (4, 33) (**Figure 1B**). These nGREs, which have been found in several metabolic and inflammatory gene promoters (e.g. IL6, IL20, STAT3), recruit nuclear receptor corepressor (NCOR1) and NCOR2 that inhibit transcription (4, 7, 27, 33, 34). Interestingly, nGRE sites were not confirmed by DNA footprinting in IL6 promoter, for instance, and are not well characterized for this gene repression by the GR agonist Dexamethasone (30, 35, 36). Conversely, GR can also bind to regions of DNA called “composite sites” that comprise both GRE and responsive elements to other TFs, and in consequence, GR interferes with these TFs. In another alternative model that explains repression, GR form protein-protein interactions with TFs (tethering), as demonstrated for NF-*κ*B, AP-1, and signal transducers and activators of transcription (STATs), ultimately reducing proinflammatory genes expression. Both mechanisms, in specific contexts, can generate inhibition or increase in the expression of certain genes (**Figure 1B**) (10, 33, 36–40). Popularized as a repression mechanism independent of DNA-binding, the classical model of tethering has been revisited, as recent experiments with cell lines have shown that GR recognize cryptic GRE sites within, or near, AP-1 or NF-κB response elements (TREs or κBRE), leading to modulation of the transactivation activity of these TFs (41). In summary, profound differences in GR modes of action will be achieved by multimeric, dimeric or monomeric forms, depending on the gene and GR binding sites (26, 42).

The physiological role of monomeric GR has been discussed for a while and little is known about its exact functioning. While discussions and evidence of a predominant role are emerging (43), others have reported that monomeric GR binds weakly to chromatin and thus cannot activate or repress genes (44), pointing out that, *in vivo*, the GR structure is predominantly dimeric. The dimerization state may present cell-specific characteristics, and may present different oligomerization states depending on the origin of the stimulus, endogenous or exogenous (45). For a deeper understanding of the mechanisms and roles of GR, we suggest dedicated reviews (7, 13, 46).

The interactions described above, which are involved in the different mechanisms mediated by GRs, can promote PS events that may play an important role in the regulation of gene expression associated with GCs.

## Phase separation

The relevance of PS for understanding signal transduction relies on a higher level of molecular interaction and function control. Also known as coacervation, PS is a spontaneous and reversible process in which intracellular molecules, such as proteins and nucleic acids, begin to interact more with each other than with the surrounding environment, forming membraneless organelles [e.g., stress granules, processing bodies (P-bodies), Cajal bodies, and nucleolus] (47–51). This is often caused by the increase in the local concentration of these molecules above a critical point, and by physical changes in the cells, such as changes in temperature or pH. When certain molecules exceed their concentration threshold, it becomes thermodynamically more favorable for them to unite and interact with each other, de-mixing until the free energy of the medium stabilizes (52). Thus, biomolecular condensates are products of this physicochemical phenomenon, and consequently, promote local changes on key molecules availability, or interfere with the stability of molecular complexes that relay or block effector signals.

**Figure 2:**
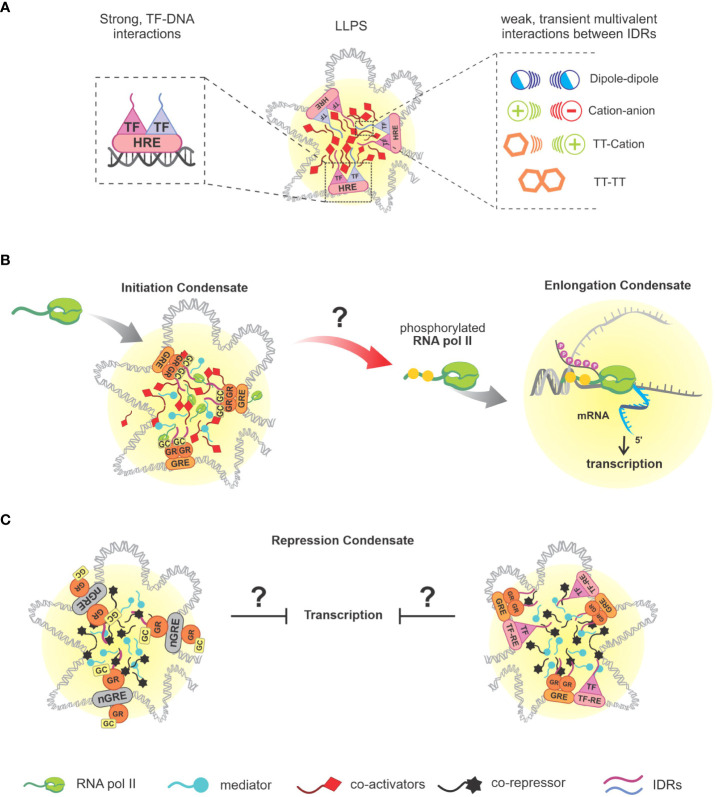
Separation of genomic phases and associated interactions. **(A)** Illustrative representation of DNA-protein condensates nucleation, followed by a representation of the interaction of SRs, profiled as TFs as a functional role, together with their HRE that mediate the beginning of phase separation, and weak interactions established by the present molecules *via* IDRs that provide stability to the condensates, which can be: dipole-dipole, cation-anion, π-cation, π-π; **(B)** Overview of GR-mediated transcriptional condensate (sGRE), based on *in vitro* study (56). It remains unclear if the recruitment of RNA pol II activity actually happens as represented, direct experimental data regarding gene expression of GR-regulated genes and LLPS are not available yet. **(C)** Illustrative representations of GR LLPS mediated by nGREs sites and composite sites, respectively, based on published data (23). SRs, steroid receptors; TFs, transcription factors; HRE, hormone response elements; IDRs, intrinsically disordered regions; GR, glucocorticoid receptor; RNA pol II, RNA polymerase II.

Most intracellular condensates are composed of proteins that have stretches of low-complexity sequences, called intrinsically disordered regions (IDRs). These regions are formed by polar and charged amino acids residues, such as glycines, serines, prolines, lysines and arginines (53, 54), and, interspersedly, have aromatic residues such as tyrosine and phenylalanine. Thus, these sequences allow proteins to make weak bonds, such as pi-pi, cation-pi, dipole-dipole or cation-anion (55), which are some of the interactions responsible for allowing the PS of protein-RNA complexes, even in a system where entropy tends to favor homogeneity (**Figure 2A**). Post- translational modifications (57, 58), binding interactions (59–62) and environmental conditions (63–65) regulate interactions between the proteins and other macromolecules in the condensate. Regarding their conformation, condensates could vary according to their composition, types of interactions and the surrounding environment, and they may appear as droplets (liquid), colloids (gel-like), fibers or crystals (solid) (66–68). LLPS rapidly emerged as a proposed model for the formation of membraneless organelles (biomolecular condensates), however, a word of caution is necessary because it has been argued that many studies superficially analyzed whether this paradigm applies for *in vivo* conditions, where other drivers of macromolecular concentration are also plausible [see refs (69, 70) for a discussion]. Nevertheless, LLPS predominates as the proposed process where molecules interact with each other through multivalent interactions promoted by IDRs, generating condensates that have liquid-like structural characteristics, such as spherical structure, high fusion power by contact and dynamism (47, 71), and because they are highly dynamic, there is a constant exchange of molecules between the condensate and its envelope. However, even though they are classified as similar, condensates will differ from their surrounding system, as LLPS products are highly viscous and have their own surface tension, resulting in dense droplets capable of melting (21, 47). Due to the high concentration of molecules inside, biomolecular condensates are related to the potentiation of intracellular reactions, such as bonds between molecules and catalysis of reactions in consequence of the concentration of enzymes and substrates. For instance, as it occurs in the carboxylation of the RuBP protein (ribulose bi- phosphate) by the enzyme Rubisco (RuBP carboxylase/oxygenase) in the carbon fixation process of photosynthesis (72, 73), or in the process of pathogen DNA recognition by cyclic guanosine monophosphate (GMP)-adenosine monophosphate (AMP) synthase (cGAS) through binding, causing increased intracellular cGAMP levels and innate immunity activation (74). Condensates may also act in the suppression of some pathways, as in RNA granules, where there is an increase in mRNA deadenylation (75). Furthermore, recent studies associate the condensates formed from LLPS to transcriptional control, promoting the activation or repression of specific genes (16, 76).

To explain the process of transcriptional regulation in which PS is involved, we first need to look at the keystone of this process: TFs. DBDs allow the recognition of enhancers or promoter sequences, and IDRs present in TFs will promote the nucleation of these complexes/condensates, generating regions of foci (77) (**Figure 2A**). Due to the surface tension generated from the nucleation of these condensates, these regions will reciprocally recognize each other and coalesce, promoting the recruitment of the Mediator complex (which mediates the communication between transcriptional activators and the transcriptional machinery) and RNA pol II. Therefore, this DNA-protein interaction defines the so-called transcriptional condensate, or initiation condensate (78–87). By transitioning from the initiation phase to the elongation phase, RNA pol II will have its C-terminal domain (CTD) phosphorylated by cyclin-dependent kinase (CDK)7 and CDK9, allowing it to separate from the initiation condensate and begin to interact with elongation factors (EFs) and with a pre-mRNA, undergoing a new PS (78, 81, 83) (**Figure 2B**). Subsequently, P-TEFb (positive transcription elongation factor b) will enter the new condensate, promoting CTD hyperphosphorylation of RNA pol II, accelerating transcription elongation. The now hyperphosphorylated RNA pol II will interact with factors present in RNA splicing regions, and as the concentration of new mRNA molecules increases, the splicing condensate breaks down and RNA pol II returns to the initiation condensate (88), [reviewed in (22, 52)]. Altogether, transcription machinery is highly dependent on LLPS events that orchestrate the molecular dynamics involved in gene expression at chromatin level. A closing thought for this subject is the idea that LLPS can promote a nuclear domain where TFs can easily find their binding sites in DNA or other proteins. For instance, Wagh et al. discuss that TFs search for DNA binding sites and random encounters would be very ineffective, a situation improved by molecular confinement in condensates. LLPS can efficiently connect enhancers and promoters and recruit the transcriptional machinery (52). Complementary, nuclear condensates may also repress gene expression (next section).

## Phase separation associated with other steroid receptors (AR, ER, MR and PR)

A growing number of proteins involved in transcription were reported to perform LLPS, such as Yes-associated protein (YAP), transcriptional co-activator with PDZ-binding motif (TAZ), TP53 and β-catenin, which are proteins associated with TFs functions involved in cell-cycle, proliferation or differentiation (87, 89, 90). Remarkably, some SRs, such as androgen receptor (AR), estrogen receptor (ER), progesterone receptor (PR), and mineralocorticoid receptor (MR) were also identified as susceptible to PS in the context of transcriptional regulation (15, 23, 52, 56, 78, 82, 91–101). This process can happen through the protagonism of different domains according to each specific SR, even though there is considerable similarity between these NRs, both in terms of structure and physico-chemical propensy to LLPS formation (56). These domains can be considered more relevant when their inhibition/ablation impairs LLPS formation, or redundant if their inhibition can only prevent LLPS in the absence of another domain (56, 97). Like GR, the majority of other SRs need IDRs to stabilize the interaction with co-regulators of this process, such as Med-1, p300/CBP, PCAF, and NCOA1 (92, 97, 102). This fact points to a common, and probably conserved, mechanism of LLPS that is critical for some of SRs functions as transcriptional regulatory molecules.

Although the majority of studies suggests that ER is a receptor that forms liquid condensate related to transcriptional regulation (52, 82, 98, 99), still a minority of others point out that most of the ER pool would not be related to the transcriptional function of the receptor (103). Likewise, GR foci was presented to be possibly non-transcriptional by a study published in 1995; however, subsequent studies indicated a plausible role of GR foci in GR’s transcriptional regulation (15, 23, 56, 104, 105). Similarly, condensates modulated by AR are described as active transcriptional regions that depend on AR’s multi-domain, a marked feature of GR LLPS and other transcription factors. The splice variant AR-v7 that lacks LBD and is incapable of forming foci reveals that this domain mediates critical interaction in AR-mediated LLPS (93, 106). Not surprisingly, PS with PR has also been proposed as a molecular event involved in transcriptional regulation, which was corroborated by evidence that PR dimers act as important functional units, similarly to what happens with GR (100). Notably, the promotion of GR LLPS has a domain-dependent profile, comparable to AR, as observed in a robust study performed by Stortz and collaborators (15). A fundamental domain is LBD, since its removal generates a noticeable decrease in GR foci formation, conversely, the absence of NTD only leads to an alteration of its stability, possibly due to the presence of IDRs in this region. It’s important to underscore that the induced monomeric structure of GR generates less foci density, and that constitutively tetrameric mutant-GR has an improved potential of forming condensates in comparison to both, monomeric and dimeric forms, indicating that there’s a dependency on quaternary structures in order to lead to a more effective foci formation. Ultimately, the similarity between the formation of these SRs’ condensates point out to a common function of these NRs’ LLPS in active transcriptional sites.

## Possible implications of GR-mediated phase separation

Since GR-LLPS has been reported only recently, experimental data is still lacking in order to determine how condensates are implicated in the different GR actions. Some of the evidence that makes plausible a GR-LLPS contribution to transcriptional regulation is the DNA-dependence on GR foci formation. In addition, GR condensates retain the ability to bind sequence specific DNA, as demonstrated by GRE-DNA probes recruitment to GR/TIF2 foci along with the presence of coactivators (15, 23, 104, 105). NCOA2 condensate (a.k.a. TIF2), containing p300 and PCAF, has been reported to be co-localized with GR condensate at its target sites (104, 105). In addition, the co-localization of GR condensates with Med-1 corroborates the suggestion that GR LLPS is involved in transcriptional regulation (15, 23). Thus far, the relationship between GR and Pol-II has been questioned due to partial colocalization evidence. Nevertheless, recent studies involving GR LLPS have indicated that there may be a transcriptional relationship between these two molecules, a subject that still needs clarification (23, 56). Altogether, the similarity between GR and other SRs, as well as NCOA2 and Med-1 participation in the generation of GR condensate, strongly suggest that GR LLPS is involved in transcriptional regulation (**Figure 2B**).

Furthermore, composite sites and nGREs are two interaction mechanisms of GR that have been recently described to improve the intensity of GR condensates *in vitro* associated with its co-regulator (i.e., G9a) when compared to canonical GRE sites. In contrast, greater increase in GR-Med-1 condensates’ intensity is a feature of presence of GREs (23). While much has been understood concerning GR LLPS involving GRE sites, the other mechanisms of GR interaction still lack information related to PS (**Figure 2C**).

Taking to account the function of GR in respect of inflammatory regulation, it is inevitable to question whether there is a relation between condensates of GR-homodimers and coactivators with GREs that promote the transcription of anti-inflammatory genes (e.g., I*κ*B*α*/*NFKBIA*, GILZ/*TSC22D3*, MKP-1/*DUSP1*), and if this process would be optimized by the formation of GR condensate. Nevertheless, this question requires a great set of studies in order to be slightly more elucidated, especially towards gene expression.

## Concluding remarks and future directions

GR regulates the expression of metabolic and proinflammatory genes through multiple mechanisms that take place in the nucleus. After binding to DNA, GR have the ability to nucleate LLPS through IDRs present in GR and its molecular partners, forming condensates that coalesce, resulting in a large DNA-protein complex. These events seem to be required for transcription of GR-dependent genes, and some of these genes promote downregulation of proinflammatory signaling pathways. GR condensates are also predicted to repress transcription. Although this repression may be inapplicable to hamper pro-inflammatory gene expression regulated by GR, it could be a relevant target for curbing GCs side-effects. Knowledge in this field will depend not only on mechanistic studies involving LLPS and transcription of GCs-responsive genes, including those related to inflammation, but also on new experimental settings that overcome the limitations of nucleus imaging and cell-free systems that only suggest liquid-like properties of manipulated condensates.

## Author contributions

EP, AP, TP, LS and IG wrote the manuscript. All authors contributed to the article and approved the submitted version.

## References

[1] Motavalli R Majidi T Pourlak T Abediazar S Shoja MM Zununi Vahed S. The clinical significance of the glucocorticoid receptors: Genetics and epigenetics. J Steroid Biochem Mol Biol (2021) 213:105952. doi: 10.1016/j.jsbmb.2021.105952 34274458

[2] Præstholm SM Correia CM Goitea VE Siersbæk MS Jørgensen M Havelund JF. Impaired glucocorticoid receptor expression in liver disrupts feeding-induced gene expression, glucose uptake, and glycogen storage. Cell Rep (2021) 37:109938. doi: 10.1016/j.celrep.2021.109938 34731602

[3] Azmi NASM Juliana N Azmani S Effendy NM Abu IF Teng NIMF. Cortisol on circadian rhythm and its effect on cardiovascular system. Int J Environ Res Public Health (2021) 18:1–15. doi: 10.3390/ijerph18020676 PMC7830980 33466883

[4] Surjit M Ganti KP Mukherji A Ye T Hua G Metzger D. Widespread negative response elements mediate direct repression by agonist-liganded glucocorticoid receptor. Cell (2011) 145:224–41. doi: 10.1016/j.cell.2011.03.027 21496643

[5] Göttlicher M Heck S Herrlich P. Transcriptional cross-talk, the second mode of steroid hormone receptor action. J Mol Med (1998) 76:480–9. doi: 10.1007/s001090050242 9660166

[6] Sinha S Rosin NL Arora R Labit E Jaffer A Cao L. Dexamethasone modulates immature neutrophils and interferon programming in severe COVID-19. Nat Med (2022) 28:201–11. doi: 10.1038/s41591-021-01576-3 PMC8799469 34782790

[7] Cain DW Cidlowski JA. Immune regulation by glucocorticoids. Nat Rev Immunol (2017) 17:233–47. doi: 10.1038/nri.2017.1 PMC9761406 28192415

[8] Group RC Horby P Lim WS Emberson JR Mafham M Bell JL. Dexamethasone in hospitalized patients with covid-19. N Engl J Med (2021) 384:693–704. doi: 10.1056/NEJMoa2021436 32678530 PMC7383595

[9] Del Valle DM Kim-Schulze S Huang H-H Beckmann ND Nirenberg S Wang B. An inflammatory cytokine signature predicts COVID-19 severity and survival. Nat Med (2020) 26:1636–43. doi: 10.1038/s41591-020-1051-9 PMC7869028 32839624

[10] Almawi WY Lipman ML Stevens AC Zanker B Hadro ET Strom TB. Abrogation of glucocorticoid-mediated inhibition of T cell proliferation by the synergistic action of IL-1, IL-6, and IFN-gamma. J Immunol (1991) 146:3523–7. doi: 10.4049/jimmunol.146.10.3523 1902856

[11] Ataie-Kachoie P Pourgholami MH Morris DL. Inhibition of the IL-6 signaling pathway: A strategy to combat chronic inflammatory diseases and cancer. Cytokine Growth Factor Rev (2013) 24:163–73. doi: 10.1016/j.cytogfr.2012.09.001 23107589

[12] Liu B Li M Zhou Z Guan X Xiang Y. Can we use interleukin-6 (IL-6) blockade for coronavirus disease 2019 (COVID-19)-induced cytokine release syndrome (CRS)? J Autoimmun (2020) 111:102452. doi: 10.1016/j.jaut.2020.102452 32291137 PMC7151347

[13] Timmermans S Souffriau J Libert C. A general introduction to glucocorticoid biology. Front Immunol (2019) 10:1545. doi: 10.3389/fimmu.2019.01545 31333672 PMC6621919

[14] Amano Y Lee SW Allison AC. Inhibition by glucocorticoids of the formation of interleukin-1 alpha, interleukin-1 beta, and interleukin-6: mediation by decreased mRNA stability. Mol Pharmacol (1993) 43:176 LP – 182. 8429822

[15] Stortz M Pecci A Presman DM Levi V. Unraveling the molecular interactions involved in phase separation of glucocorticoid receptor. BMC Biol (2020) 18:1–20. doi: 10.1186/s12915-020-00788-2 32487073 PMC7268505

[16] O’Connell LC Mowry KL. Regulation of spatially restricted gene expression: Linking RNA localization and phase separation. Biochem Soc Trans (2021) 49:2591–600. doi: 10.1042/BST20210320 PMC8939473 34821361

[17] Peng P-H Hsu K-W Wu K-J. Liquid-liquid phase separation (LLPS) in cellular physiology and tumor biology. Am J Cancer Res (2021) 11:3766–76. PMC8414392 34522448

[18] Wang B Zhang L Dai T Qin Z Lu H Zhang L. Liquid-liquid phase separation in human health and diseases. Signal Transduct Target Ther (2021) 6:290. doi: 10.1038/s41392-021-00678-1 34334791 PMC8326283

[19] Gomes E Shorter J. The molecular language of membraneless organelles. J Biol Chem (2019) 294:7115–27. doi: 10.1074/jbc.TM118.001192 PMC6509512 30045872

[20] Peng A Weber SC. Evidence for and against liquid-liquid phase separation in the nucleus. Non-coding RNA (2019) 5(4):50. doi: 10.3390/ncrna5040050 PMC6958436 31683819

[21] Shin Y Brangwynne CP. Liquid phase condensation in cell physiology and disease. Science (2017) 357(6357):eaaf4382. doi: 10.1126/science.aaf4382 28935776

[22] Peng L Li EM Xu LY. From start to end: Phase separation and transcriptional regulation. Biochim Biophys Acta - Gene Regul Mech (2020) 1863:194641. doi: 10.1016/j.bbagrm.2020.194641 33017669

[23] Frank F Liu X Ortlund EA. Glucocorticoid receptor condensates link DNA-dependent receptor dimerization and transcriptional transactivation. Proc Natl Acad Sci U.S.A. (2021) 118(30):e2024685118. doi: 10.1073/pnas.2024685118 34285072 PMC8325269

[24] Nicolaides NC Galata Z Kino T Chrousos GP Charmandari E. The human glucocorticoid receptor: molecular basis of biologic function. Steroids (2010) 75:1–12. doi: 10.1016/j.steroids.2009.09.002 19818358 PMC2813911

[25] Lu NZ Cidlowski JA. Glucocorticoid receptor isoforms generate transcription specificity. Trends Cell Biol (2006) 16:301–7. doi: 10.1016/j.tcb.2006.04.005 16697199

[26] Presman DM Ganguly S Schiltz RL Johnson TA Karpova TS Hager GL. DNA Binding triggers tetramerization of the glucocorticoid receptor in live cells. Proc Natl Acad Sci U.S.A. (2016) 113:8236–41. doi: 10.1073/pnas.1606774113 PMC4961135 27382178

[27] Chakravarti D LaMorte VJ Nelson MC Nakajima T Schulman IG Juguilon H. Role of CBP/P300 in nuclear receptor signalling. Nature (1996) 383:99–103. doi: 10.1038/383099a0 8779723

[28] Nicolaides NC Roberts ML Kino T Braatvedt G Hurt DE Katsantoni E. A novel point mutation of the human glucocorticoid receptor gene causes primary generalized glucocorticoid resistance through impaired interaction with the LXXLL motif of the p160 coactivators: Dissociation of the transactivating and transreppressive acti. J Clin Endocrinol Metab (2014) 99:902–7. doi: 10.1210/jc.2013-3005 PMC4010692 24483153

[29] Weikum ER Knuesel MT Ortlund EA Yamamoto KR. Glucocorticoid receptor control of transcription: precision and plasticity *via* allostery. Nat Rev Mol Cell Biol (2017) 18:159–74. doi: 10.1038/nrm.2016.152 PMC6257982 28053348

[30] Rao NAS McCalman MT Moulos P Francoijs K-J Chatziioannou A Kolisis FN. Coactivation of GR and NFKB alters the repertoire of their binding sites and target genes. Genome Res (2011) 21:1404–16. doi: 10.1101/gr.118042.110 PMC3166826 21750107

[31] Ehrchen JM Roth J Barczyk-Kahlert K. More than suppression: Glucocorticoid action on monocytes and macrophages. Front Immunol (2019) 10:2028. doi: 10.3389/fimmu.2019.02028 31507614 PMC6718555

[32] Oakley RH Cidlowski JA. The biology of the glucocorticoid receptor: new signaling mechanisms in health and disease. J Allergy Clin Immunol (2013) 132:1033–44. doi: 10.1016/j.jaci.2013.09.007 PMC4084612 24084075

[33] Hudson WH Youn C Ortlund EA. The structural basis of direct glucocorticoid-mediated transrepression. Nat Struct Mol Biol (2013) 20:53–8. doi: 10.1038/nsmb.2456 PMC3539207 23222642

[34] Newton R Shah S Altonsy MO Gerber AN. Glucocorticoid and cytokine crosstalk: Feedback, feedforward, and co-regulatory interactions determine repression or resistance. J Biol Chem (2017) 292:7163–72. doi: 10.1074/jbc.R117.777318 PMC5409484 28283576

[35] Ray A LaForge KS Sehgal PB. On the mechanism for efficient repression of the interleukin-6 promoter by glucocorticoids: Enhancer, TATA box, and RNA start site (Inr motif) occlusion. Mol Cell Biol (1990) 10:5736–46. doi: 10.1128/mcb.10.11.5736-5746.1990 PMC361346 2233715

[36] Ray A Prefontaine KE. Physical association and functional antagonism between the p65 subunit of transcription factor NF-kappa b and the glucocorticoid receptor. Proc Natl Acad Sci (1994) 91:752–6. doi: 10.1073/pnas.91.2.752 PMC43027 8290595

[37] Xavier AM Anunciato AKO Rosenstock TR Glezer I. Gene expression control by glucocorticoid receptors during innate immune responses. Front Endocrinol (Lausanne) (2016) 7:31. doi: 10.3389/fendo.2016.00031 27148162 PMC4835445

[38] Miyata M Lee J-Y Susuki-Miyata S Wang WY Xu H Kai H. Glucocorticoids suppress inflammation *via* the upregulation of negative regulator IRAK-m. Nat Commun (2015) 6:6062. doi: 10.1038/ncomms7062 25585690 PMC4309435

[39] Beck IME Vanden Berghe W Vermeulen L Bougarne N Vander Cruyssen B Haegeman G. Altered subcellular distribution of MSK1 induced by glucocorticoids contributes to NF-κB inhibition. EMBO J (2008) 27:1682–93. doi: 10.1038/emboj.2008.95 PMC2435130 18511904

[40] Strickland BA Ansari SA Dantoft W Uhlenhaut NH. How to tame your genes: mechanisms of inflammatory gene repression by glucocorticoids. FEBS Lett (2022) 596:2596–616. doi: 10.1002/1873-3468.14409 35612756

[41] Hudson WH de Vera IMS Nwachukwu JC Weikum ER Herbst AG Yang Q. Cryptic glucocorticoid receptor-binding sites pervade genomic NF-κB response elements. Nat Commun (2018) 9:1337. doi: 10.1038/s41467-018-03780-1 29626214 PMC5889392

[42] Jiménez-Panizo A Alegre-Martí A Tettey TT Fettweis G Abella M Antón R. The multivalency of the glucocorticoid receptor ligand-binding domain explains its manifold physiological activities. Nucleic Acids Res (2022) 50:13063–82. doi: 10.1093/nar/gkac1119 PMC9825158 36464162

[43] Timmermans S Verhoog NJD Van Looveren K Dewaele S Hochepied T Eggermont M. Point mutation I634A in the glucocorticoid receptor causes embryonic lethality by reduced ligand binding. J Biol Chem (2022) 298:101574. doi: 10.1016/j.jbc.2022.101574 35007536 PMC8808175

[44] Johnson TA Paakinaho V Kim S Hager GL Presman DM. Genome-wide binding potential and regulatory activity of the glucocorticoid receptor’s monomeric and dimeric forms. Nat Commun (2021) 12:1–14. doi: 10.1038/s41467-021-22234-9 33790284 PMC8012360

[45] Vettorazzi S Nalbantoglu D Gebhardt JCM Tuckermann J. A guide to changing paradigms of glucocorticoid receptor function-a model system for genome regulation and physiology. FEBS J (2021) 289(19):5718–43. doi: 10.1111/febs.16100 34213830

[46] Ayroldi E Libert C Kiemer AK. Glucocorticoids in immunity and inflammation. Front Media SA (2021), 5718–5743. doi: 10.3389/978-2-88971-743-9

[47] Banani SF Lee HO Hyman AA Rosen MK. Biomolecular condensates: Organizers of cellular biochemistry. Nat Rev Mol Cell Biol (2017) 18:285–98. doi: 10.1038/nrm.2017.7 PMC7434221 28225081

[48] Brangwynne CP Eckmann CR Courson DS Rybarska A Hoege C Gharakhani J. Germline p granules are liquid droplets that localize by controlled dissolution/condensation. Science (2009) 324:1729–32. doi: 10.1126/science.1172046 19460965

[49] Handwerger KE Cordero JA Gall JG. Cajal bodies, nucleoli, and speckles in the xenopus oocyte nucleus have a low-density, sponge-like structure. Mol Biol Cell (2005) 16:202–11. doi: 10.1091/mbc.E04 PMC539164 15509651

[50] Hernandez-Verdun D. Assembly and disassembly of the nucleolus during the cell cycle. Nucleus (2011) 2:189–94. doi: 10.4161/nucl.2.3.16246 PMC3149879 21818412

[51] Li P Banjade S Cheng HC Kim S Chen B Guo L. Phase transitions in the assembly of multivalent signalling proteins. Nature (2012) 483:336–40. doi: 10.1038/nature10879 PMC3343696 22398450

[52] Wagh K Garcia DA Upadhyaya A. Phase separation in transcription factor dynamics and chromatin organization. Curr Opin Struct Biol (2021) 71:148–55. doi: 10.1016/j.sbi.2021.06.009 PMC8794578 34303933

[53] Romero P Obradovic Z Li X Garner EC Brown CJ Dunker AK. Sequence complexity of disordered protein - Romero - 2000 - proteins: Structure, function, and bioinformatics. Wiley Online Libr (2001) 48:38–48. doi: 10.1002/1097-0134(20010101)42:1<38::aid-prot50>3.0.co;2-3 11093259

[54] Vucetic S Brown CJ Dunker AK Obradovic Z. Flavors of protein disorder. Proteins Struct Funct Genet (2003) 52:573–84. doi: 10.1002/prot.10437 12910457

[55] Brangwynne CP Tompa P Pappu RV. Polymer physics of intracellular phase transitions. Nat Phys (2015) 11:899–904. doi: 10.1038/nphys3532

[56] Stortz M Presman DM Pecci A Levi V. Phasing the intranuclear organization of steroid hormone receptors. Biochem J (2021) 478:443–61. doi: 10.1042/BCJ20200883 33512446

[57] Banani SF Rice AM Peeples WB Lin Y Jain S Parker R. Compositional control of phase-separated cellular bodies. Cell (2016) 166:651–63. doi: 10.1016/j.cell.2016.06.010 PMC4967043 27374333

[58] Nott TJ Petsalaki E Farber P Jervis D Fussner E Plochowietz A. Phase transition of a disordered nuage protein generates environmentally responsive membraneless organelles. Mol Cell (2015) 57:936–47. doi: 10.1016/j.molcel.2015.01.013 PMC4352761 25747659

[59] Guo L Kim HJ Wang H Monaghan J Freyermuth F Sung JC. Nuclear-import receptors reverse aberrant phase transitions of RNA-binding proteins with prion-like domains. Cell (2018) 173:677–692.e20. doi: 10.1016/j.cell.2018.03.002 29677512 PMC5911940

[60] Hofweber M Hutten S Bourgeois B Spreitzer E Niedner-Boblenz A Schifferer M. Phase separation of FUS is suppressed by its nuclear import receptor and arginine methylation. Cell (2018) 173:706–719.e13. doi: 10.1016/j.cell.2018.03.004 29677514

[61] Qamar S Wang GZ Randle SJ Ruggeri FS Varela JA Lin JQ. FUS phase separation is modulated by a molecular chaperone and methylation of arginine cation-π interactions. Cell (2018) 173:720–734.e15. doi: 10.1016/j.cell.2018.03.056 29677515 PMC5927716

[62] Yoshizawa T Ali R Jiou J Fung HYJ Burke KA Kim SJ. Nuclear import receptor inhibits phase separation of FUS through binding to multiple sites. Cell (2018) 173:693–705.e22. doi: 10.1016/j.cell.2018.03.003 29677513 PMC6234985

[63] Franzmann TM Jahnel M Pozniakovsky A Mahamid J Holehouse AS Nüske E. Phase separation of a yeast prion protein promotes cellular fitness. Science (2018) 359(6371):eaao5654. doi: 10.1126/science.aao5654 29301985

[64] Kato M Yang YS Sutter BM Wang Y McKnight SL Tu BP. Redox state controls phase separation of the yeast ataxin-2 protein *via* reversible oxidation of its methionine-rich low-complexity domain. Cell (2019) 177:711–721.e8. doi: 10.1016/j.cell.2019.02.044 30982603 PMC6752730

[65] Riback JA Katanski CD Kear-Scott JL Pilipenko EV Rojek AE Sosnick TR. Stress-triggered phase separation is an adaptive, evolutionarily tuned response. Cell (2017) 168:1028–1040.e19. doi: 10.1016/j.cell.2017.02.027 28283059 PMC5401687

[66] Hyman AA Weber CA Jülicher F. Liquid-liquid phase separation in biology. Annu Rev Cell Dev Biol (2014) 30:39–58. doi: 10.1146/annurev-cellbio-100913-013325 25288112

[67] Vernon RMC Chong PA Tsang B Kim TH Bah A Farber P. Pi-pi contacts are an overlooked protein feature relevant to phase separation. Elife (2018) 7:1–48. doi: 10.7554/eLife.31486 PMC5847340 29424691

[68] Wang J Choi JM Holehouse AS Lee HO Zhang X Jahnel M. A molecular grammar governing the driving forces for phase separation of prion-like RNA binding proteins. Cell (2018) 174:688–699.e16. doi: 10.1016/j.cell.2018.06.006 29961577 PMC6063760

[69] Musacchio A. On the role of phase separation in the biogenesis of membraneless compartments. EMBO J (2022) 41:e109952. doi: 10.15252/embj.2021109952 35107832 PMC8886532

[70] McSwiggen DT Mir M Darzacq X Tjian R. Evaluating phase separation in live cells: diagnosis, caveats, and functional consequences. Genes Dev (2019) 33:1619–34. doi: 10.1101/gad.331520.119 PMC6942051 31594803

[71] Alberti S Hyman AA. Biomolecular condensates at the nexus of cellular stress, protein aggregation disease and ageing. Nat Rev Mol Cell Biol (2021) 22:196–213. doi: 10.1038/s41580-020-00326-6 33510441

[72] Andersson I Backlund A. Structure and function of rubisco. Plant Physiol Biochem (2008) 46:275–91. doi: 10.1016/j.plaphy.2008.01.001 18294858

[73] Zang K Wang H Hartl FU Hayer-Hartl M. Scaffolding protein CcmM directs multiprotein phase separation in β-carboxysome biogenesis. Nat Struct Mol Biol (2021) 28:909–22. doi: 10.1038/s41594-021-00676-5 PMC8580825 34759380

[74] Du M Chen ZJ. DNA-Induced liquid phase condensation of cGAS activates innate immune signaling. Science (2018) 361:704–9. doi: 10.1126/science.aat1022 PMC9417938 29976794

[75] Sheu-Gruttadauria J MacRae IJ. Phase transitions in the assembly and function of human miRISC. Cell (2018) 173:946–957.e16. doi: 10.1016/j.cell.2018.02.051 29576456 PMC5935535

[76] Zhang H Ji X Li P Liu C Lou J Wang Z. Liquid-liquid phase separation in biology: mechanisms, physiological functions and human diseases. Sci China Life Sci (2020) 63:953–85. doi: 10.1007/s11427-020-1702-x 32548680

[77] Wei MT Chang YC Shimobayashi SF Shin Y Strom AR Brangwynne CP. Nucleated transcriptional condensates amplify gene expression. Nat Cell Biol (2020) 22:1187–96. doi: 10.1038/s41556-020-00578-6 32929202

[78] Boija A Klein IA Sabari BR Dall’Agnese A Coffey EL Zamudio AV. Transcription factors activate genes through the phase-separation capacity of their activation domains. Cell (2018) 175:1842–1855.e16. doi: 10.1016/j.cell.2018.10.042 30449618 PMC6295254

[79] Cai D Feliciano D Dong P Flores E Gruebele M Porat-Shliom N. Phase separation of YAP reorganizes genome topology for long-term YAP target gene expression. Nat Cell Biol (2019) 21:1578–89. doi: 10.1038/s41556-019-0433-z PMC8259329 31792379

[80] Cho WK Spille JH Hecht M Lee C Li C Grube V. Mediator and RNA polymerase II clusters associate in transcription-dependent condensates. Science (2018) 361:412–5. doi: 10.1126/science.aar4199 PMC6543815 29930094

[81] Chong S Dugast-Darzacq C Liu Z Dong P Dailey GM Cattoglio C. Imaging dynamic and selective low-complexity domain interactions that control gene transcription. Science (2018) 361(6400):eaar2555. doi: 10.1126/science.aar2555 29930090 PMC6961784

[82] Nair SJ Yang L Meluzzi D Oh S Yang F Friedman MJ. Phase separation of ligand-activated enhancers licenses cooperative chromosomal enhancer assembly. Nat Struct Mol Biol (2019) 26:193–203. doi: 10.1038/s41594-019-0190-5 30833784 PMC6709854

[83] Sabari BR Dall’Agnese A Boija A Klein IA Coffey EL Shrinivas K. Coactivator condensation at super-enhancers links phase separation and gene control. Science (2018) 361(6400):eaar3958. doi: 10.1126/science.aar3958 29930091 PMC6092193

[84] Shin Y Chang YC Lee DSW Berry J Sanders DW Ronceray P. Liquid nuclear condensates mechanically sense and restructure the genome. Cell (2018) 175:1481–1491.e13. doi: 10.1016/j.cell.2018.10.057 30500535 PMC6724728

[85] Soutourina J. Transcription regulation by the mediator complex. Nat Rev Mol Cell Biol (2018) 19:262–74. doi: 10.1038/nrm.2017.115 29209056

[86] Whyte WA Orlando DA Hnisz D Abraham BJ Lin CY Kagey MH. Master transcription factors and mediator establish super-enhancers at key cell identity genes. Cell (2013) 153:307–19. doi: 10.1016/j.cell.2013.03.035 PMC3653129 23582322

[87] Zamudio AV Dall’Agnese A Henninger JE Manteiga JC Afeyan LK Hannett NM. Mediator condensates localize signaling factors to key cell identity genes. Mol Cell (2019) 76:753–766.e6. doi: 10.1016/j.molcel.2019.08.016 31563432 PMC6898777

[88] Guo YE Manteiga JC Henninger JE Sabari BR Shrinivas K Abraham BJ. Pol II phosphorylation regulates a switch between transcriptional and splicing condensates. Nature (2019) 572:543. doi: 10.1038/s41586-019-1464-0 31391587 PMC6706314

[89] Yu M Peng Z Qin M Liu Y Wang J Zhang C. Interferon-γ induces tumor resistance to anti-PD-1 immunotherapy by promoting YAP phase separation. Mol Cell (2021) 81:1216–1230.e9. doi: 10.1016/j.molcel.2021.01.010 33606996

[90] Xu J Reumers J Couceiro JR De Smet F Gallardo R Rudyak S. Gain of function of mutant p53 by coaggregation with multiple tumor suppressors. Nat Chem Biol (2011) 7:285–95. doi: 10.1038/nchembio.546 21445056

[91] Pearce D Náray-Fejes-Tóth A Fejes-Tóth G. Determinants of subnuclear organization of mineralocorticoid receptor characterized through analysis of wild type and mutant receptors *. J Biol Chem (2002) 277:1451–6. doi: 10.1074/jbc.M105966200 11677231

[92] Frank F Ortlund EA Liu X. Structural insights into glucocorticoid receptor function. Biochem Soc Trans (2021) 49:2333–43. doi: 10.1042/BST20210419 PMC9274455 34709368

[93] Ahmed J Meszaros A Lazar T Tompa P. DNA-Binding domain as the minimal region driving RNA-dependent liquid–liquid phase separation of androgen receptor. Protein Sci (2021) 30:1380–92. doi: 10.1002/pro.4100 PMC8197421 33938068

[94] Ban F Leblanc E Cavga AD Huang C-CF Flory MR Zhang F. Development of an androgen receptor inhibitor targeting the n-terminal domain of androgen receptor for treatment of castration resistant prostate cancer. Cancers (2021) 13(14):3488. doi: 10.3390/cancers13143488 PMC8304761 34298700

[95] Bielskutė S Garcia-Cabau C Frigolé-Vivas M Szulc E De Mol E Pesarrodona M. Low amounts of heavy water increase the phase separation propensity of a fragment of the androgen receptor activation domain. Protein Sci (2021) 30:1427–37. doi: 10.1002/pro.4110 PMC8197436 33978290

[96] Roggero CM Esser V Duan L Rice AM Ma S Raj GV. Poly-glutamine-dependent self-association as a potential mechanism for regulation of androgen receptor activity. PloS One (2022) 17:e0258876. doi: 10.1371/journal.pone.0258876 34986150 PMC8730435

[97] Więch A Tarczewska A Ożyhar A Orłowski M. Metal ions induce liquid condensate formation by the f domain of aedes aegypti ecdysteroid receptor. New Perspect Nucl Receptor Stud Cells (2021) 10(3):571. doi: 10.3390/cells10030571 PMC7999165 33807814

[98] Saravanan B Soota D Islam Z Majumdar S Mann R Meel S. Ligand dependent gene regulation by transient ERα clustered enhancers. PloS Genet (2020) 16:e1008516. doi: 10.1371/journal.pgen.1008516 31905229 PMC6975561

[99] Tsang B Pritišanac I Scherer SW Moses AM Forman-Kay JD. Phase separation as a missing mechanism for interpretation of disease mutations. Cell (2020) 183:1742–56. doi: 10.1016/j.cell.2020.11.050 33357399

[100] Arnett-Mansfield RL Graham JD Hanson AR Mote PA Gompel A Scurr LL. Focal subnuclear distribution of progesterone receptor is ligand dependent and associated with transcriptional activity. Mol Endocrinol (2007) 21:14–29. doi: 10.1210/me.2006-0041 17021053

[101] Graham JD Hanson AR Croft AJ Fox AH Clarke CL. Nuclear matrix binding is critical for progesterone receptor movement into nuclear foci. FASEB J (2009) 23:546–56. doi: 10.1096/fj.08-113639 18931262

[102] Sołtys K Ożyhar A. Transcription regulators and membraneless organelles challenges to investigate them. Int J Mol Sci (2021) 22(23):12758. doi: 10.3390/ijms222312758 34884563 PMC8657783

[103] Stenoien DL Mancini MG Patel K Allegretto EA Smith CL Mancini MA. Subnuclear trafficking of estrogen receptor-α and steroid receptor coactivator-1. Mol Endocrinol (2000) 14:518–34. doi: 10.1210/mend.14.4.0436 10770489

[104] Stortz M Presman DM Bruno L Annibale P Dansey MV Burton G. Mapping the dynamics of the glucocorticoid receptor within the nuclear landscape. Sci Rep (2017) 7(23):12758. doi: 10.1038/s41598-017-06676-0 PMC5524710 28740156

[105] Ogawa H Yu RT Haraguchi T Hiraoka Y Nakatani Y Morohashi K. Nuclear structure-associated TIF2 recruits glucocorticoid receptor and its target DNA. Biochem Biophys Res Commun (2004) 320:218–25. doi: 10.1016/j.bbrc.2004.05.161 15207724

[106] Zhang F Biswas M Massah S Lee J Lingadahalli S Wong S. Dynamic phase separation of the androgen receptor and its coactivators key to regulate gene expression. Nucleic Acids Res (2023) 51:99–116. doi: 10.1093/nar/gkac1158 36535377 PMC9841400

